# Supplementary material for: Disruption of small RNAs and mechanistic variation in Segregation Distorter: A sperm-killing drive system in Drosophila melanogaster
Source: PLoS Genet. 2026 Aug 3;22(8):e1012235. doi: 10.1371/journal.pgen.1012235 (PMC13460739; doi:10.1371/journal.pgen.1012235)
Supplement: Supplementary file 1 [file pgen.1012235.s001.pdf]

Supplemental Table 1

Genotype	*SD* chromosome	*Rsp^s* chromosome	male transmission			female transmission	
			k_m ±SEM	k_m* ±SEM	*n*	k_f ±SEM	*n*
SD-5/Gla	*SD-5*	*Gla*	1 ±0.000	1 ±0.000	1515	0.518 ±0.017	685
SD-5/Iso-1	*SD-5*	*Iso-1*	1 ±0.000	1 ±0.000	1057	0.533 ±0.025	973
SD-Mad/Gla	*SD-Mad*	*Gla*	0.875 ±0.024	0.848 ±0.029	1026	0.560 ±0.020	473
SD-Mad/Iso-1	*SD-Mad*	*Iso-1*	0.984 ±0.005	0.981 ±0.006	1633	0.549 ±0.012	1548
SD-Mad^Rev/Iso-1	*SD-Mad^Rev*	*Iso-1*	0.582 ±0.012	0.541 ±0.012	1346	0.541 ±0.011	884

Supplemental Table 2

	SD-Mad				SD-MadRev		SD-5			
	Iso1		Gla		Iso1		Iso1		Gla	
	Log2FC	p.adj	Log2FC	p.adj	Log2FC	p.adj	Log2FC	p.adj	Log2FC	p.adj
1.688	-1.10	4.78E-14	-0.28	0.001	-0.63	1.01E-17	-0.71	0.024	0.05	0.489
Rsp	-0.95	5.09E-09	-0.68	2.57E-14	-0.21	0.214	-0.07	0.796	0.33	2.32E-11
20A	-0.05	0.558	0.39	7.29E-08	0.02	0.612	-0.14	0.491	0.23	3.83E-06
flamenco	-0.18	0.023	-0.14	0.066	-0.14	0.048	-0.45	9.82E-08	-0.30	4.53E-13
42AB	-0.08	0.425	-0.45	1.20E-11	-0.15	0.020	-0.16	0.187	0.07	0.343
38C1	0.22	0.020	0.16	0.060	-0.05	0.499	-0.03	0.773	-0.09	0.085
38C2	0.60	1.02E-12	0.44	4.69E-09	0.49	1.49E-08	0.64	0.000	0.34	1.24E-13
80F	-0.22	0.100	-0.37	0.003	-0.27	1.70E-05	0.08	0.770	0.01	0.888

Supplemental Table 3

	**SD-Mad**				**SD-5**			
	**Iso1**		**Gla**		**Iso1**		**Gla**	
	**Log2FC**	**p.adj**	**Log2FC**	**p.adj**	**Log2FC**	**p.adj**	**Log2FC**	**p.adj**
1.688	-0.19	1.60E-07	-0.19	1.91E-05	0.32	2.05E-03	-0.08	0.149
Rsp	-0.04	0.922	0.85	4.10E-03	-0.89	0.146	0.09	0.821
20A	0.35	0.327	-0.09	0.658	0.90	0.117	-0.22	0.557
flamenco	0.37	0.031	1.40E-03	0.994	-0.28	0.429	-0.37	0.26
42AB	-0.20	0.280	0.47	0.170	1.85	0.000	-0.01	0.980
38C1	0.26	0.196	0.08	0.829	0.11	0.371	-0.50	0.041
38C2	0.33	0.001	-0.01	0.99	0.30	0.241	-0.77	0.037
80F	0.31	0.196	0.98	1.37E-13	0.35	0.340	0.42	5.42E-07

Supplemental Table 4

**Module**	**Comparison**	**Difference**	**P.Adj**
Module A	*SD5-R16*	0.3798	0.001
Module A	*SDMad-R16*	0.8058	1.18E-05
Module A	*SDMad-SD5*	0.4260	0.0005
Module B	*SD5-R16*	-0.7354	3.79E-06
Module B	*SDMad-R16*	-0.6629	7.20E-06
Module B	*SDMad-SD5*	0.0725	0.245
Module C	*SD5-R16*	-0.6931	2.61E-05
Module C	*SDMad-R16*	0.0109	0.976
Module C	*SDMad-SD5*	0.7039	2.38E-05
Module D	*SD5-R16*	0.0756	0.970
Module D	*SDMad-R16*	-0.1565	0.878
Module D	*SDMad-SD5*	-0.2321	0.757
Module E	*SD5-R16*	0.3470	0.496
Module E	*SDMad-R16*	0.3559	0.480

Module E	*SDMad-SD5*	0.0089	0.999
Module F	*SD5-R16*	-0.2334	0.036
Module F	*SDMad-R16*	0.5445	0.0006
Module F	*SDMad-SD5*	0.7779	7.69E-05
Module G	*SD5-R16*	0.0241	0.921
Module G	*SDMad-R16*	-0.6825	8.19E-05
Module G	*SDMad-SD5*	-0.7065	6.71E-05
Module H	*SD5-R16*	0.8122	7.32E-07
Module H	*SDMad-R16*	0.4346	2.12E-05
Module H	*SDMad-SD5*	-0.3776	4.86E-05
Module I	*SD5-R16*	-0.0790	0.969
Module I	*SDMad-R16*	-0.0192	0.998
Module I	*SDMad-SD5*	0.0598	0.982

Supplemental Table 5

	**SD-Mad**				**SD-5**			
	**RspOE**		**RspOE-dsRed**		**RspOE**		**RspOE-dsRed**	
	**Log2FC**	**p.adj**	**Log2FC**	**p.adj**	**Log2FC**	**p.adj**	**Log2FC**	**p.adj**
1.688	-0.01	0.903	0.04	0.706	0.30	0.028	0.10	0.157
Rsp	0.45	1.58E-10	0.59	1.49E-26	0.47	0.009	0.43	0.008
20A	0.04	0.706	0.04	0.386	-0.18	0.027	-0.20	0.004
flamenco	0.01	0.855	0.01	0.911	-0.06	0.497	-0.14	0.011
42AB	0.02	0.706	0.01	0.911	0.48	0.353	0.64	0.005
38C1	-0.34	2.34E-11	-0.85	1.49E-217	-0.37	4.29E-05	-0.53	2.48E-12
38C2	-0.04	0.685	-0.07	0.027	-0.08	0.253	-0.12	0.018
80F	0.06	0.855	-0.06	0.892	0.28	0.431	0.37	0.030
