# Supplementary material for: Disruption of small RNAs and mechanistic variation in Segregation Distorter: A sperm-killing drive system in Drosophila melanogaster
Source: PLoS Genet. 2026 Aug 3;22(8):e1012235. doi: 10.1371/journal.pgen.1012235 (PMC13460739; doi:10.1371/journal.pgen.1012235)
Supplement: Supplementary file 2 [file pgen.1012235.s002.pdf]

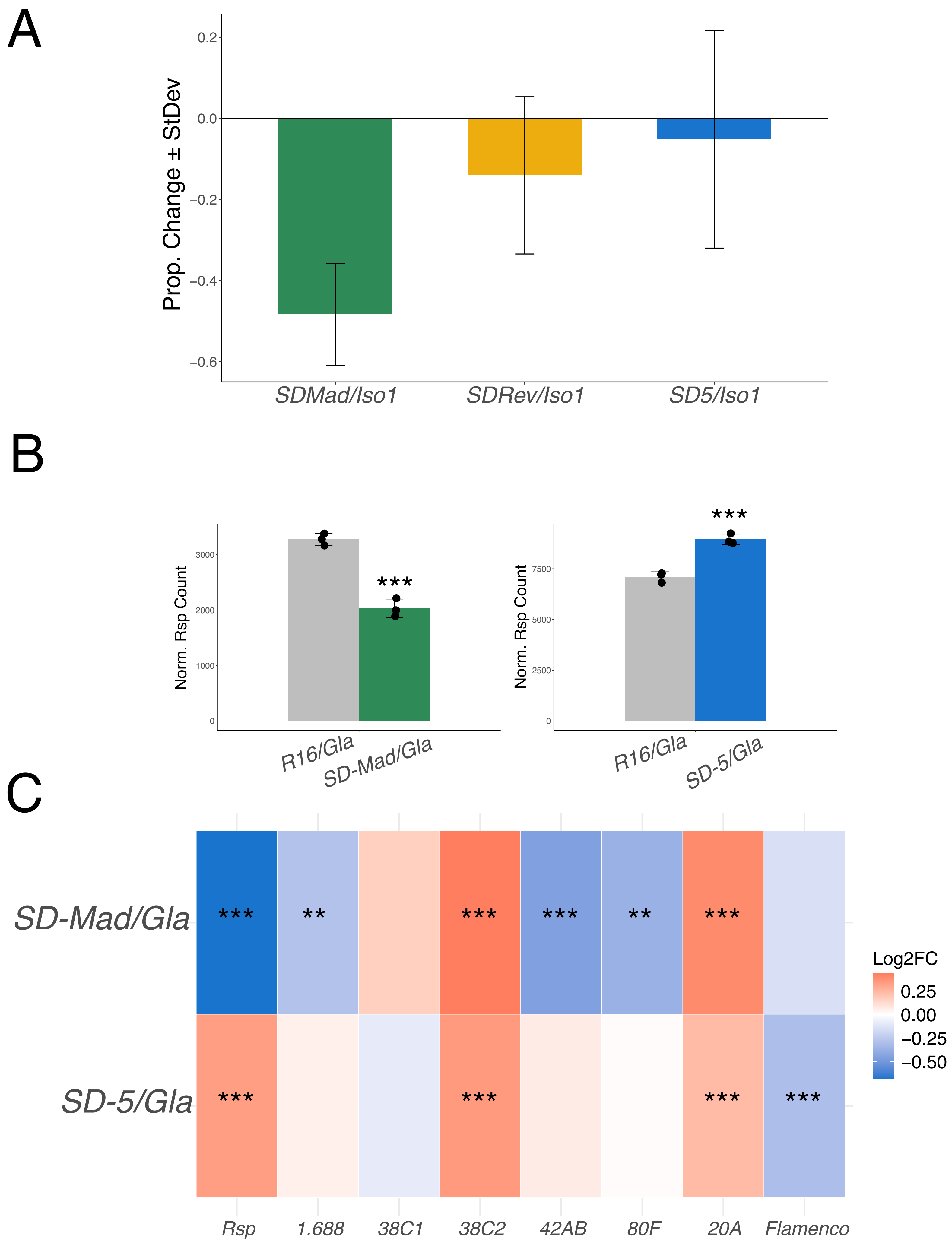
A
Prop. Change ± StDev
0.2
0.0
−0.2
−0.4
−0.6
SDMad/Iso1
SDRev/Iso1
SD5/Iso1
B
Norm. Rsp Count
3000
2000
1000
0
***
R16/Gla
SD-Mad/Gla
Norm. Rsp Count
7500
5000
2500
0
***
R16/Gla
SD-5/Gla
C
SD-Mad/Gla
*** ** *** *** ** ***
SD-5/Gla
*** *** *** ***
Rsp
1.688
38C1
38C2
42AB
80F
20A
Flamenco
Log2FC
0.25
0.00
−0.25
−0.50
