# Supplementary material for: Disruption of small RNAs and mechanistic variation in Segregation Distorter: A sperm-killing drive system in Drosophila melanogaster
Source: PLoS Genet. 2026 Aug 3;22(8):e1012235. doi: 10.1371/journal.pgen.1012235 (PMC13460739; doi:10.1371/journal.pgen.1012235)
Supplement: Supplementary file 3 [file pgen.1012235.s003.pdf]

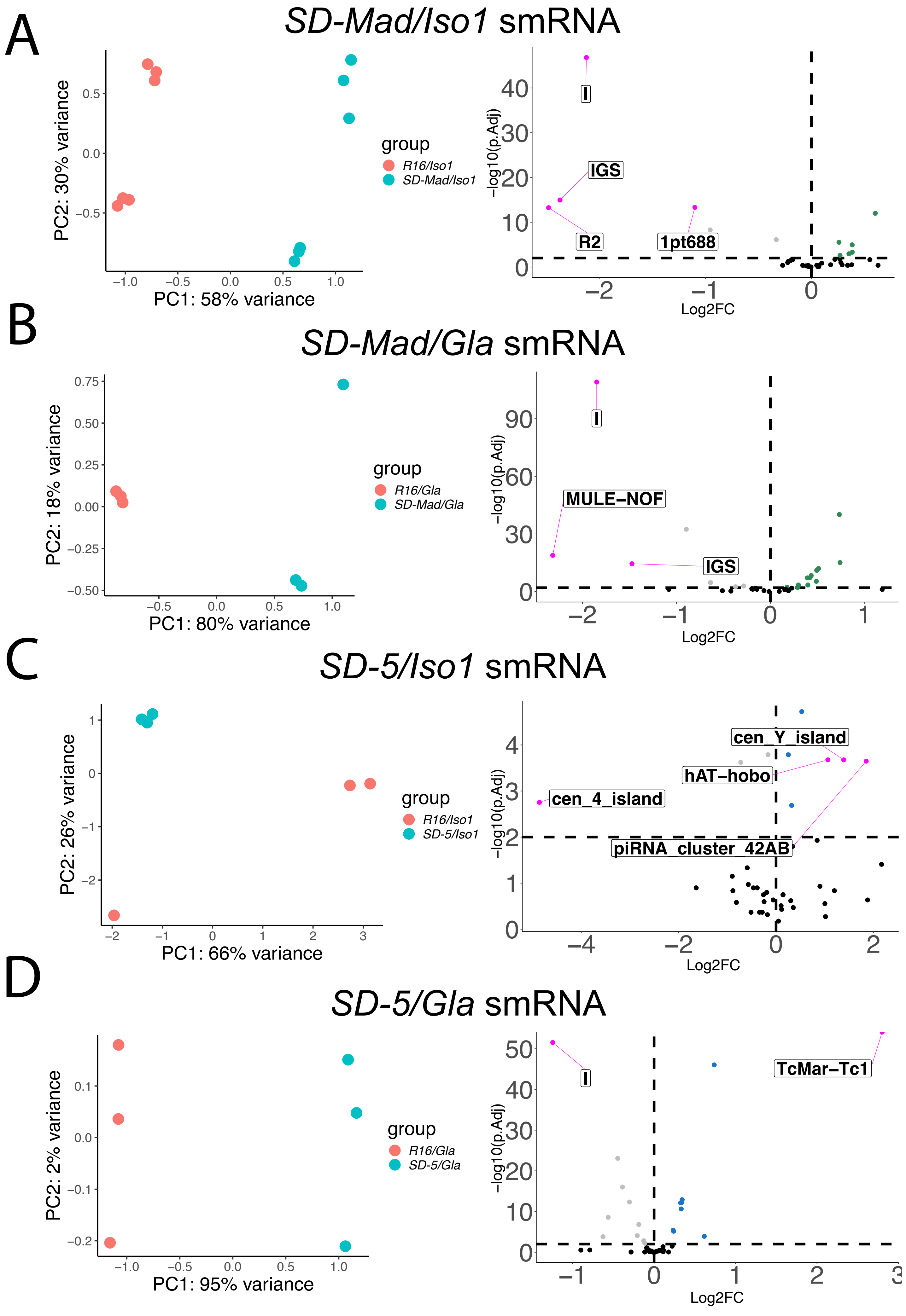
A
SD-Mad/Iso1 smRNA
PC2: 30% variance
PC1: 58% variance
group
R16/Iso1
SD-Mad/Iso1
-log10(p.Adj)
Log2FC
I
IGS
R2
1pt688
B
SD-Mad/Gla smRNA
PC2: 18% variance
PC1: 80% variance
group
R16/Gla
SD-Mad/Gla
-log10(p.Adj)
Log2FC
I
MULE-NOF
IGS
C
SD-5/Iso1 smRNA
PC2: 26% variance
PC1: 66% variance
group
R16/Iso1
SD-5/Iso1
-log10(p.Adj)
Log2FC
cen_Y_island
hAT-hobo
cen_4_island
piRNA_cluster_42AB
D
SD-5/Gla smRNA
PC2: 2% variance
PC1: 95% variance
group
R16/Gla
SD-5/Gla
-log10(p.Adj)
Log2FC
I
TcMar-Tc1
