# Supplementary material for: Disruption of small RNAs and mechanistic variation in Segregation Distorter: A sperm-killing drive system in Drosophila melanogaster
Source: PLoS Genet. 2026 Aug 3;22(8):e1012235. doi: 10.1371/journal.pgen.1012235 (PMC13460739; doi:10.1371/journal.pgen.1012235)
Supplement: Supplementary file 4 [file pgen.1012235.s004.pdf]

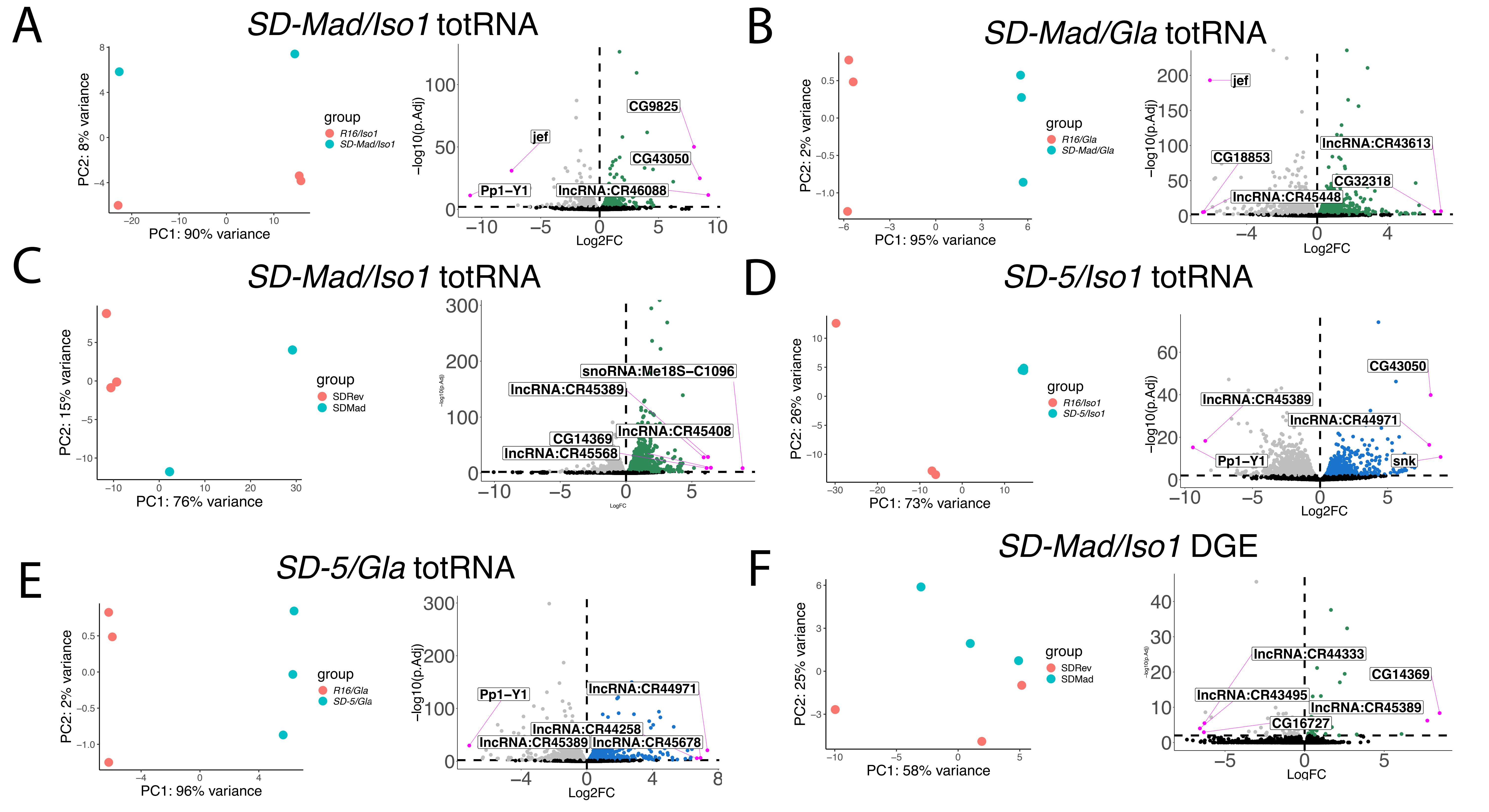
A
SD-Mad/Iso1 totRNA
PC1: 90% variance
PC2: 8% variance
group
R16/Iso1
SD-Mad/Iso1
-log10(p.Adj)
Log2FC
CG9825
jef
CG43050
Pp1-Y1
lncRNA:CR46088
B
SD-Mad/Gla totRNA
PC1: 95% variance
PC2: 2% variance
group
R16/Gla
SD-Mad/Gla
jef
CG18853
lncRNA:CR43613
CG32318
lncRNA:CR45448
C
SD-Mad/Iso1 totRNA
PC1: 76% variance
PC2: 15% variance
group
SDRev
SDMad
snoRNA:Me18S-C1096
lncRNA:CR45389
lncRNA:CR45408
CG14369
lncRNA:CR45568
D
SD-5/Iso1 totRNA
PC1: 73% variance
PC2: 26% variance
group
R16/Iso1
SD-5/Iso1
CG43050
lncRNA:CR45389
lncRNA:CR44971
Pp1-Y1
snk
E
SD-5/Gla totRNA
PC1: 96% variance
PC2: 2% variance
group
R16/Gla
SD-5/Gla
Pp1-Y1
lncRNA:CR44971
lncRNA:CR44258
lncRNA:CR45389
lncRNA:CR45678
F
SD-Mad/Iso1 DGE
PC1: 58% variance
PC2: 25% variance
group
SDRev
SDMad
LogFC
lncRNA:CR44333
CG14369
lncRNA:CR43495
lncRNA:CR45389
CG16727
