# Supplementary material for: Disruption of small RNAs and mechanistic variation in Segregation Distorter: A sperm-killing drive system in Drosophila melanogaster
Source: PLoS Genet. 2026 Aug 3;22(8):e1012235. doi: 10.1371/journal.pgen.1012235 (PMC13460739; doi:10.1371/journal.pgen.1012235)
Supplement: Supplementary file 5 [file pgen.1012235.s005.pdf]

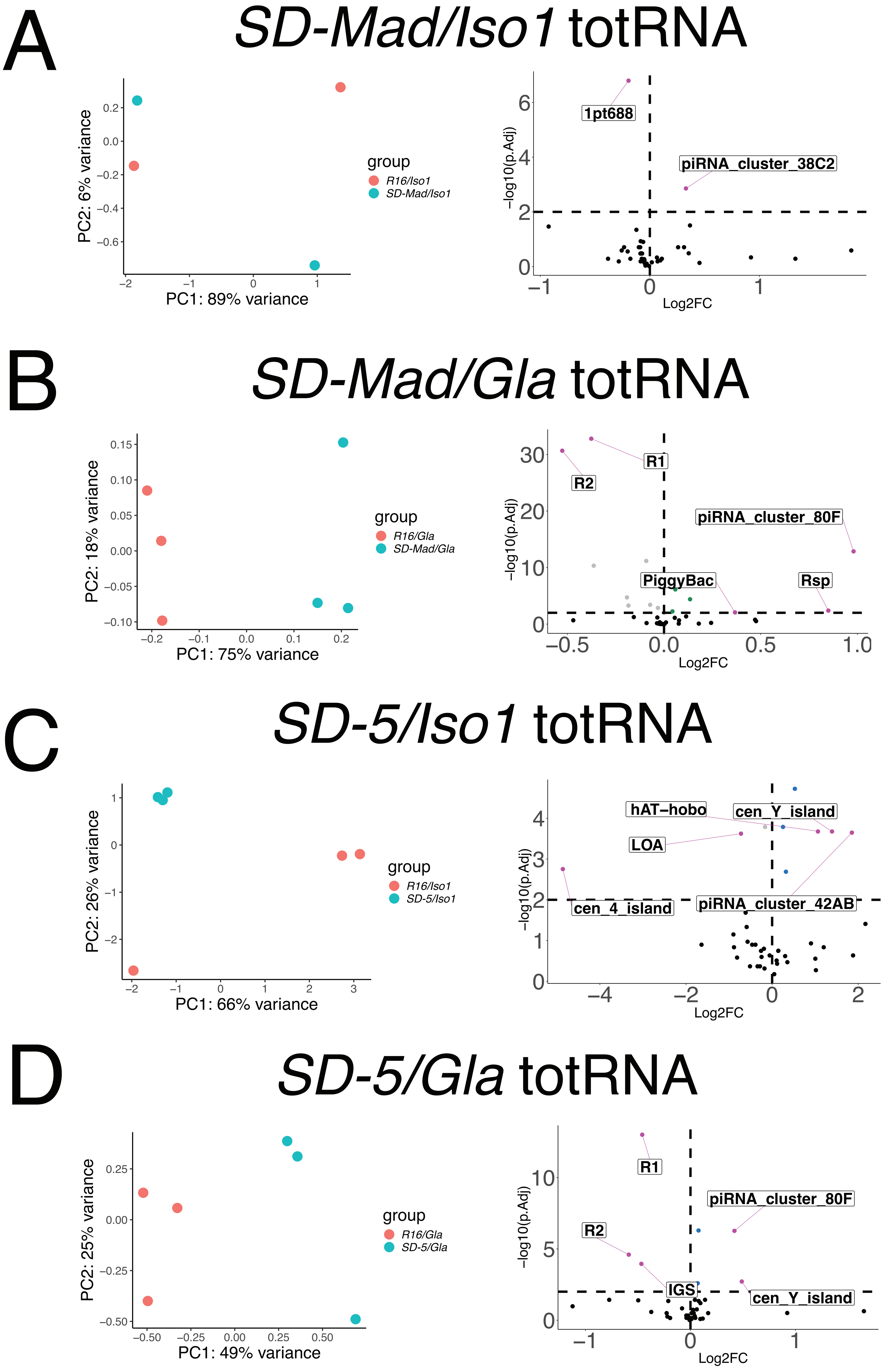

A
SD-Mad/Iso1 totRNA
PC1: 89% variance
PC2: 6% variance
group
R16/Iso1
SD-Mad/Iso1
1pt688
piRNA_cluster_38C2
Log2FC
−log10(p.Adj)
B
SD-Mad/Gla totRNA
PC1: 75% variance
PC2: 18% variance
group
R16/Gla
SD-Mad/Gla
R1
R2
piRNA_cluster_80F
PiggyBac
Rsp
Log2FC
−log10(p.Adj)
C
SD-5/Iso1 totRNA
PC1: 66% variance
PC2: 26% variance
group
R16/Iso1
SD-5/Iso1
hAT−hobo
cen_Y_island
LOA
cen_4_island
piRNA_cluster_42AB
Log2FC
−log10(p.Adj)
D
SD-5/Gla totRNA
PC1: 49% variance
PC2: 25% variance
group
R16/Gla
SD-5/Gla
R1
piRNA_cluster_80F
R2
IGS
cen_Y_island
Log2FC
−log10(p.Adj)
