# Supplementary material for: Disruption of small RNAs and mechanistic variation in Segregation Distorter: A sperm-killing drive system in Drosophila melanogaster
Source: PLoS Genet. 2026 Aug 3;22(8):e1012235. doi: 10.1371/journal.pgen.1012235 (PMC13460739; doi:10.1371/journal.pgen.1012235)
Supplement: Supplementary file 6 [file pgen.1012235.s006.pdf]

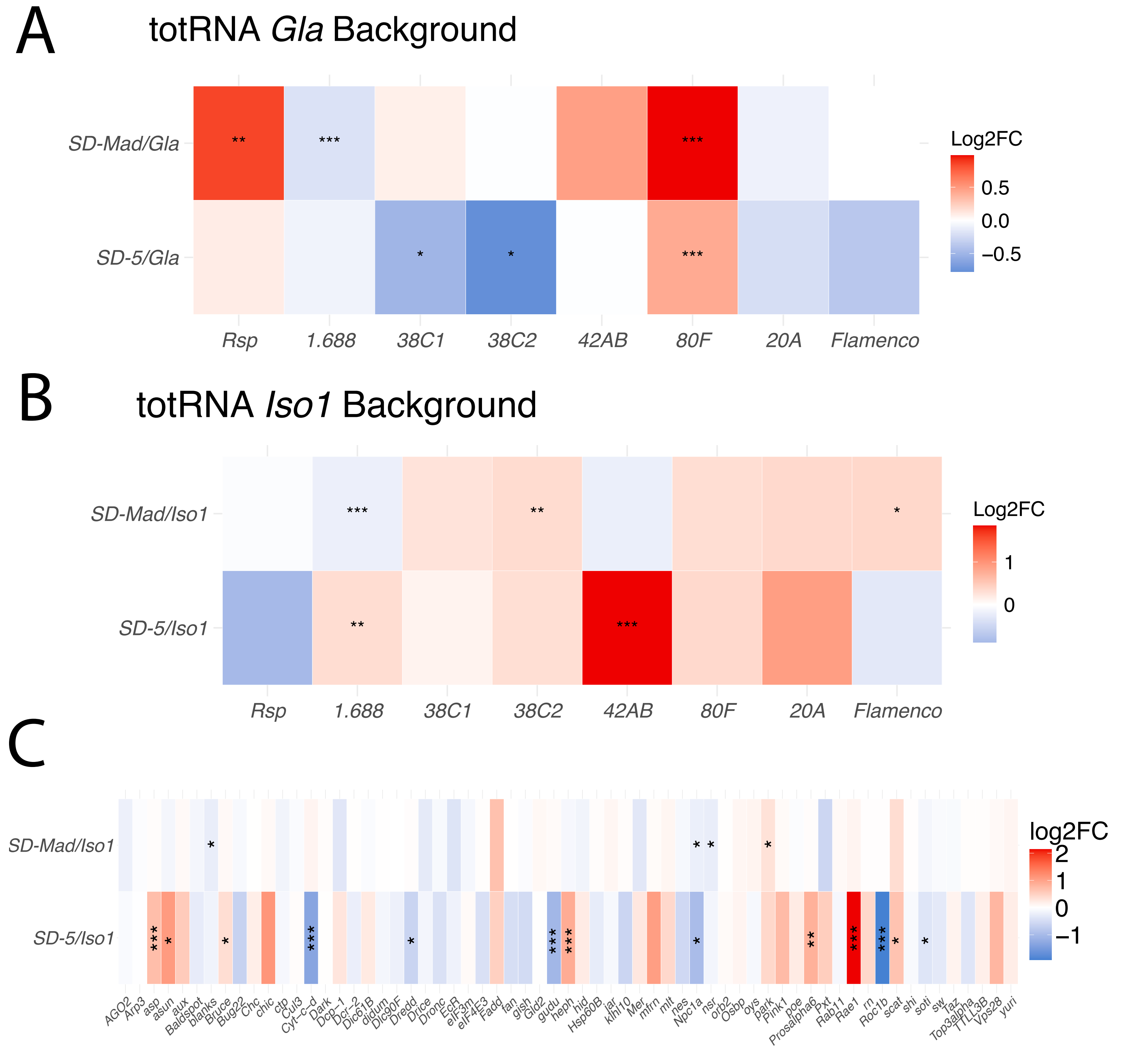
A
totRNA Gla Background
SD-Mad/Gla
SD-5/Gla
Rsp
1.688
38C1
38C2
42AB
80F
20A
Flamenco
Log2FC
0.5
0.0
−0.5
B
totRNA Iso1 Background
SD-Mad/Iso1
SD-5/Iso1
Rsp
1.688
38C1
38C2
42AB
80F
20A
Flamenco
Log2FC
1
0
C
SD-Mad/Iso1
SD-5/Iso1
AGO2
Arp3
asp
asun
aux
Baldspot
blanks
Bruce
Bug22
Chc
chic
ctp
Cul3
Cyt-c-d
Dark
Dcp-1
Dcr-2
Dic61B
didum
Dlc90F
Dredd
Drice
Dronc
EcR
eIF3m
eIF4E3
Fadd
fan
gish
Gld2
gudu
heph
hid
Hsp60B
jar
klhl10
Mer
mfrn
mlt
nes
Npc1a
nsr
orb2
Osbp
oys
park
Pink1
poe
Prosalpha6
Pxt
Rab11
Rae1
rn
Roc1b
scat
shi
soti
sw
Taz
Top3alpha
TTLL3B
Vps28
yuri
log2FC
2
1
0
−1
