# Supplementary material for: Disruption of small RNAs and mechanistic variation in Segregation Distorter: A sperm-killing drive system in Drosophila melanogaster
Source: PLoS Genet. 2026 Aug 3;22(8):e1012235. doi: 10.1371/journal.pgen.1012235 (PMC13460739; doi:10.1371/journal.pgen.1012235)
Supplement: Supplementary file 7 [file pgen.1012235.s007.pdf]

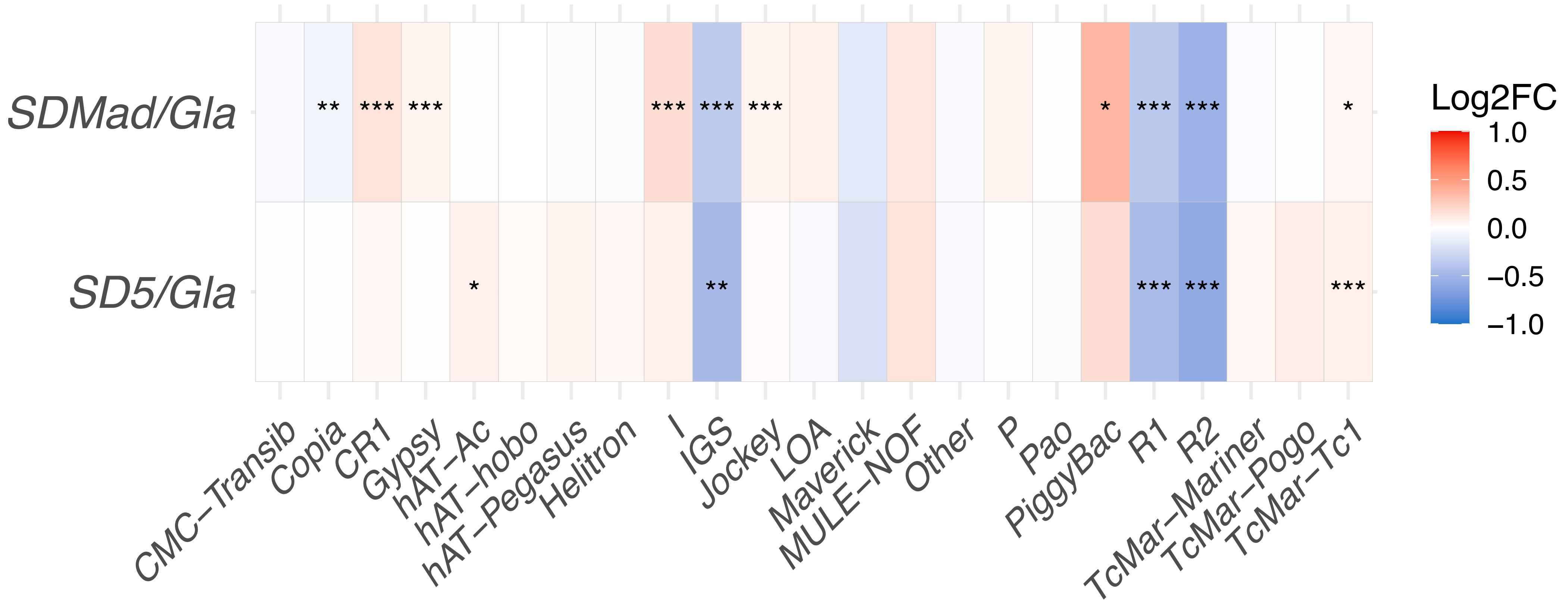

SDMad/Gla
SD5/Gla
** *** *** *** *** *** * *** *** *
* ** *** *** ***
Log2FC
1.0
0.5
0.0
−0.5
−1.0
CMC−Transib
Copia
CR1
Gypsy
hAT−Ac
hAT−hobo
hAT−Pegasus
Helitron
I
IGS
Jockey
LOA
Maverick
MULE−NOF
Other
P
Pao
PiggyBac
R1
R2
TcMar−Mariner
TcMar−Pogo
TcMar−Tc1
