# Supplementary material for: Disruption of small RNAs and mechanistic variation in Segregation Distorter: A sperm-killing drive system in Drosophila melanogaster
Source: PLoS Genet. 2026 Aug 3;22(8):e1012235. doi: 10.1371/journal.pgen.1012235 (PMC13460739; doi:10.1371/journal.pgen.1012235)
Supplement: Supplementary file 8 [file pgen.1012235.s008.pdf]

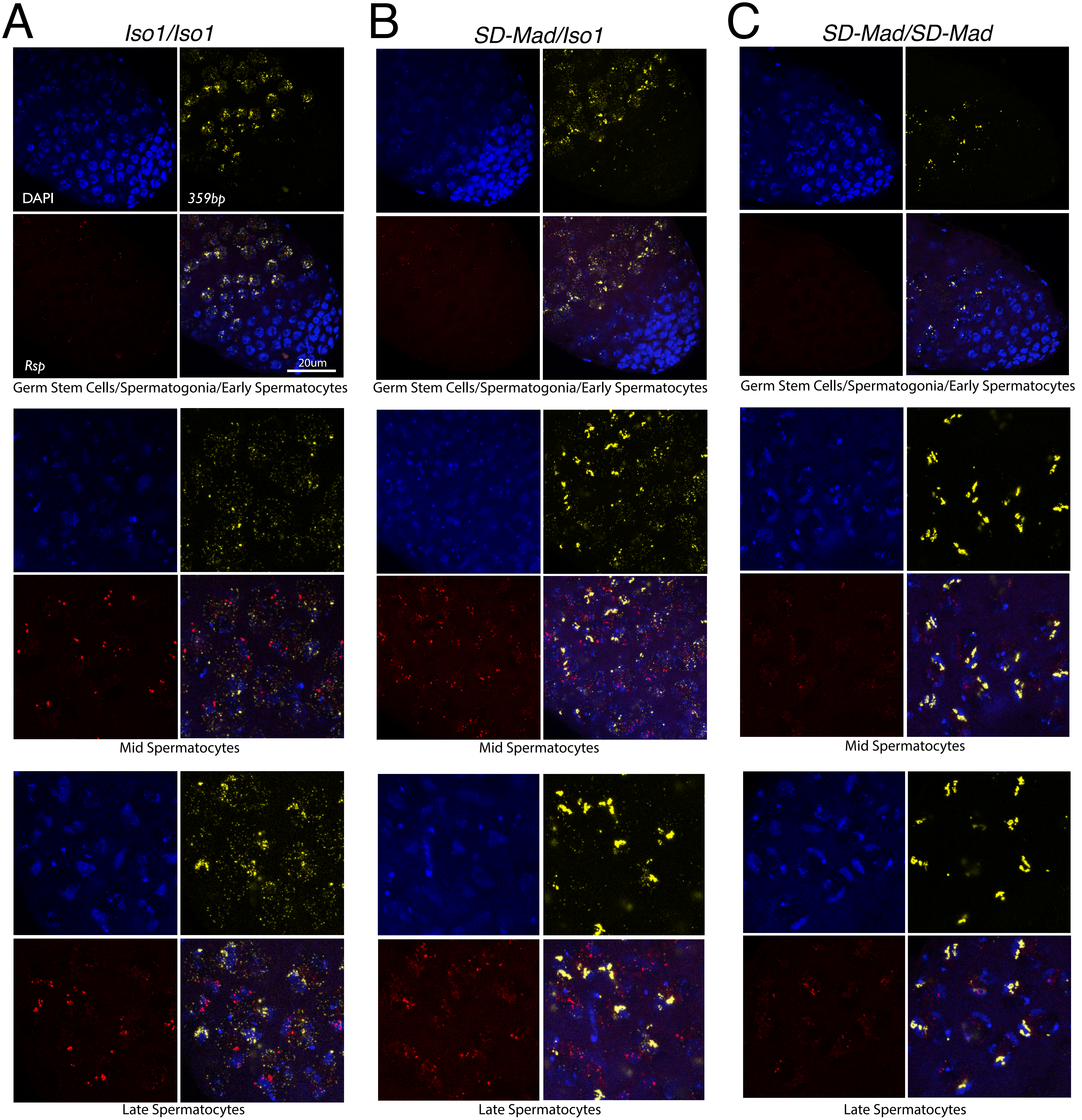
A
Iso1/Iso1
DAPI
359bp
Rsp
20um
Germ Stem Cells/Spermatogonia/Early Spermatocytes
Mid Spermatocytes
Late Spermatocytes
B
SD-Mad/Iso1
Germ Stem Cells/Spermatogonia/Early Spermatocytes
Mid Spermatocytes
Late Spermatocytes
C
SD-Mad/SD-Mad
Germ Stem Cells/Spermatogonia/Early Spermatocytes
Mid Spermatocytes
Late Spermatocytes
