# Supplementary material for: Disruption of small RNAs and mechanistic variation in Segregation Distorter: A sperm-killing drive system in Drosophila melanogaster
Source: PLoS Genet. 2026 Aug 3;22(8):e1012235. doi: 10.1371/journal.pgen.1012235 (PMC13460739; doi:10.1371/journal.pgen.1012235)
Supplement: Supplementary file 9 [file pgen.1012235.s009.pdf]

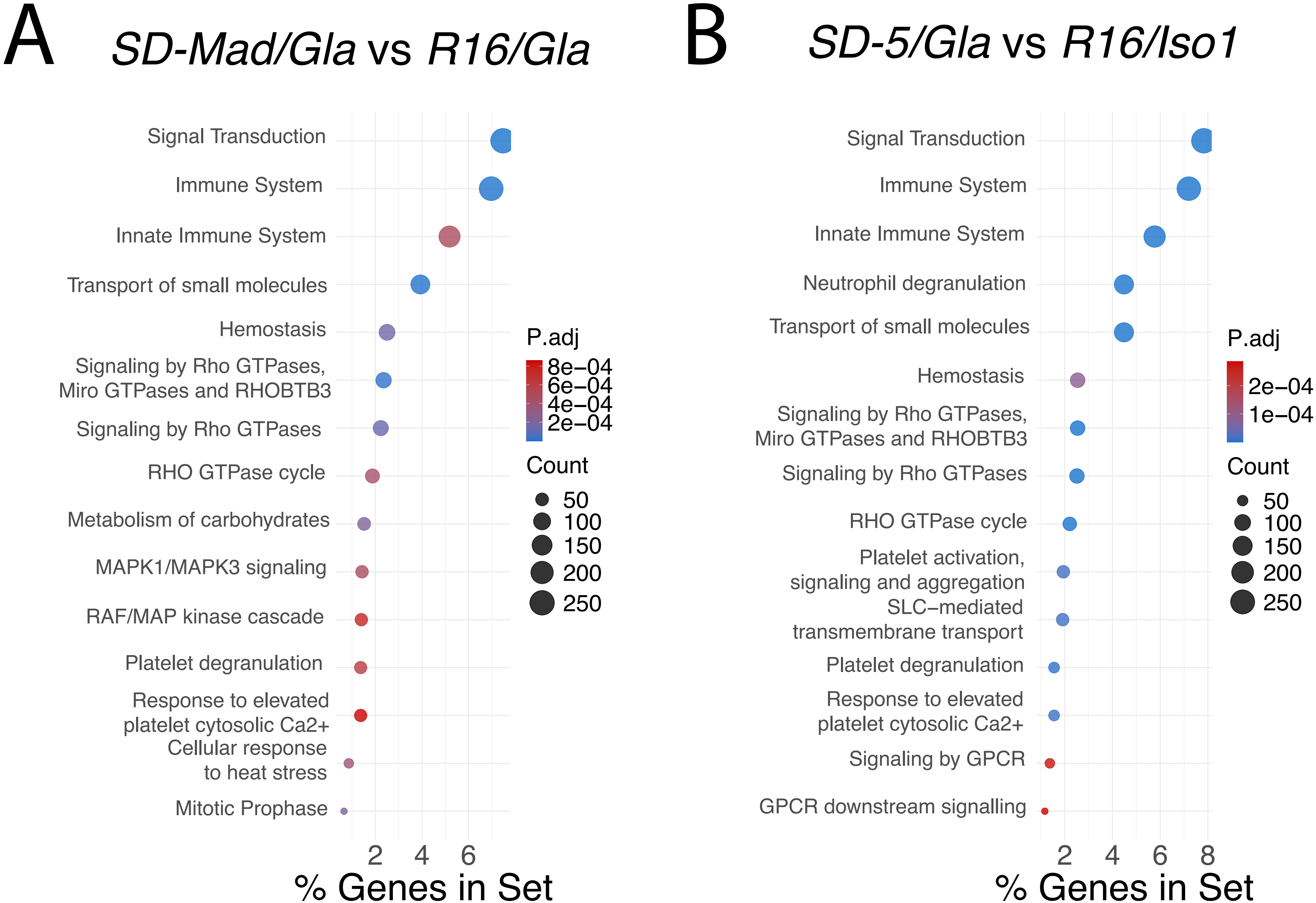
A
SD-Mad/Gla vs R16/Gla
Signal Transduction
Immune System
Innate Immune System
Transport of small molecules
Hemostasis
Signaling by Rho GTPases, Miro GTPases and RHOBTB3
Signaling by Rho GTPases
RHO GTPase cycle
Metabolism of carbohydrates
MAPK1/MAPK3 signaling
RAF/MAP kinase cascade
Platelet degranulation
Response to elevated platelet cytosolic Ca2+
Cellular response to heat stress
Mitotic Prophase
2
4
6
% Genes in Set
P.adj
8e−04
6e−04
4e−04
2e−04
Count
50
100
150
200
250
B
SD-5/Gla vs R16/Iso1
Signal Transduction
Immune System
Innate Immune System
Neutrophil degranulation
Transport of small molecules
Hemostasis
Signaling by Rho GTPases, Miro GTPases and RHOBTB3
Signaling by Rho GTPases
RHO GTPase cycle
Platelet activation, signaling and aggregation
SLC−mediated transmembrane transport
Platelet degranulation
Response to elevated platelet cytosolic Ca2+
Signaling by GPCR
GPCR downstream signalling
2
4
6
8
% Genes in Set
P.adj
2e−04
1e−04
Count
50
100
150
200
250
