# Supplementary material for: Disruption of small RNAs and mechanistic variation in Segregation Distorter: A sperm-killing drive system in Drosophila melanogaster
Source: PLoS Genet. 2026 Aug 3;22(8):e1012235. doi: 10.1371/journal.pgen.1012235 (PMC13460739; doi:10.1371/journal.pgen.1012235)
Supplement: Supplementary file 10 [file pgen.1012235.s010.pdf]

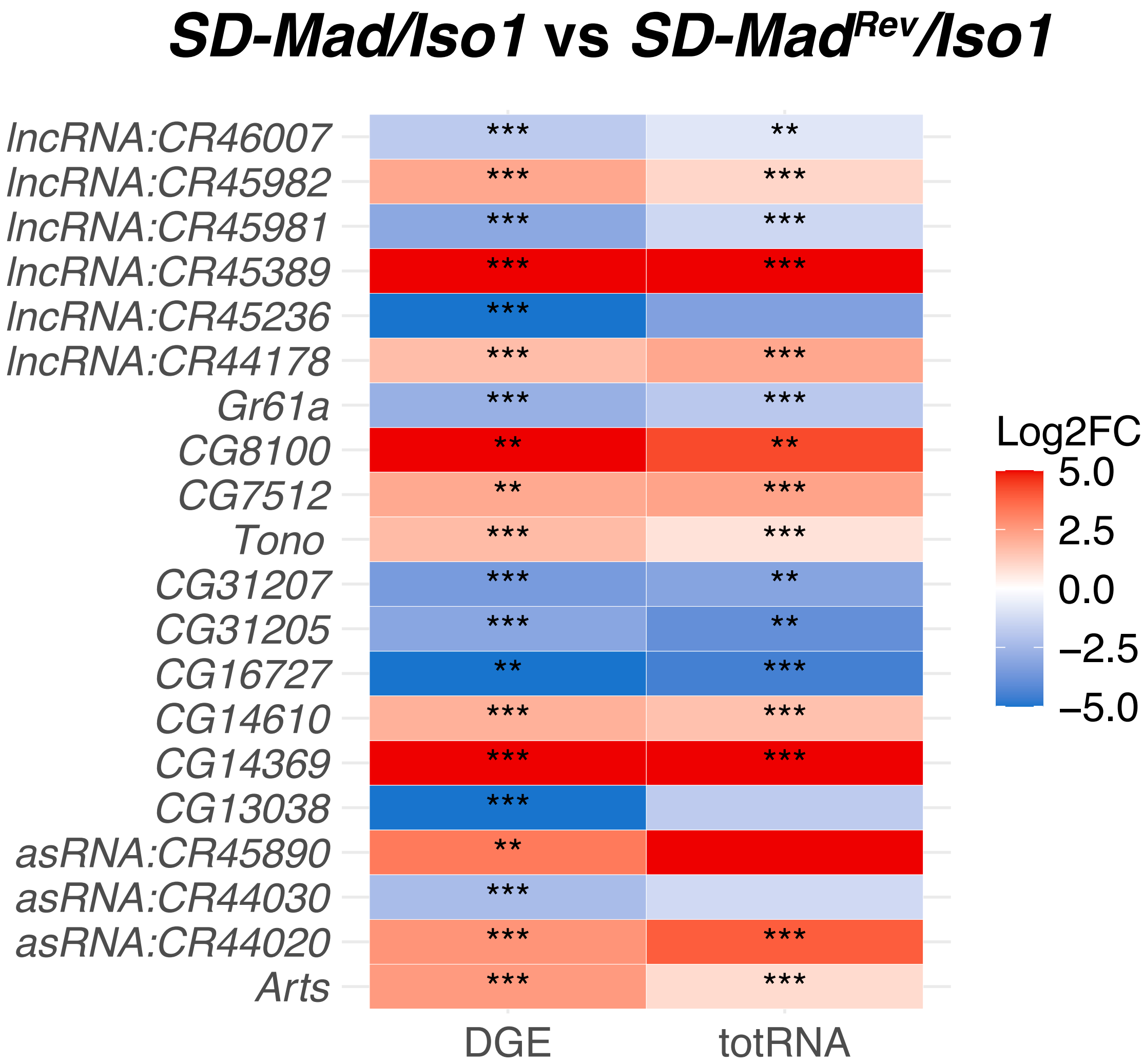

SD-Mad/Iso1 vs SD-Mad^Rev/Iso1
lncRNA:CR46007
lncRNA:CR45982
lncRNA:CR45981
lncRNA:CR45389
lncRNA:CR45236
lncRNA:CR44178
Gr61a
CG8100
CG7512
Tono
CG31207
CG31205
CG16727
CG14610
CG14369
CG13038
asRNA:CR45890
asRNA:CR44030
asRNA:CR44020
Arts
DGE
totRNA
Log2FC
5.0
2.5
0.0
−2.5
−5.0
