# Supplementary material for: Disruption of small RNAs and mechanistic variation in Segregation Distorter: A sperm-killing drive system in Drosophila melanogaster
Source: PLoS Genet. 2026 Aug 3;22(8):e1012235. doi: 10.1371/journal.pgen.1012235 (PMC13460739; doi:10.1371/journal.pgen.1012235)
Supplement: Supplementary file 11 [file pgen.1012235.s011.pdf]

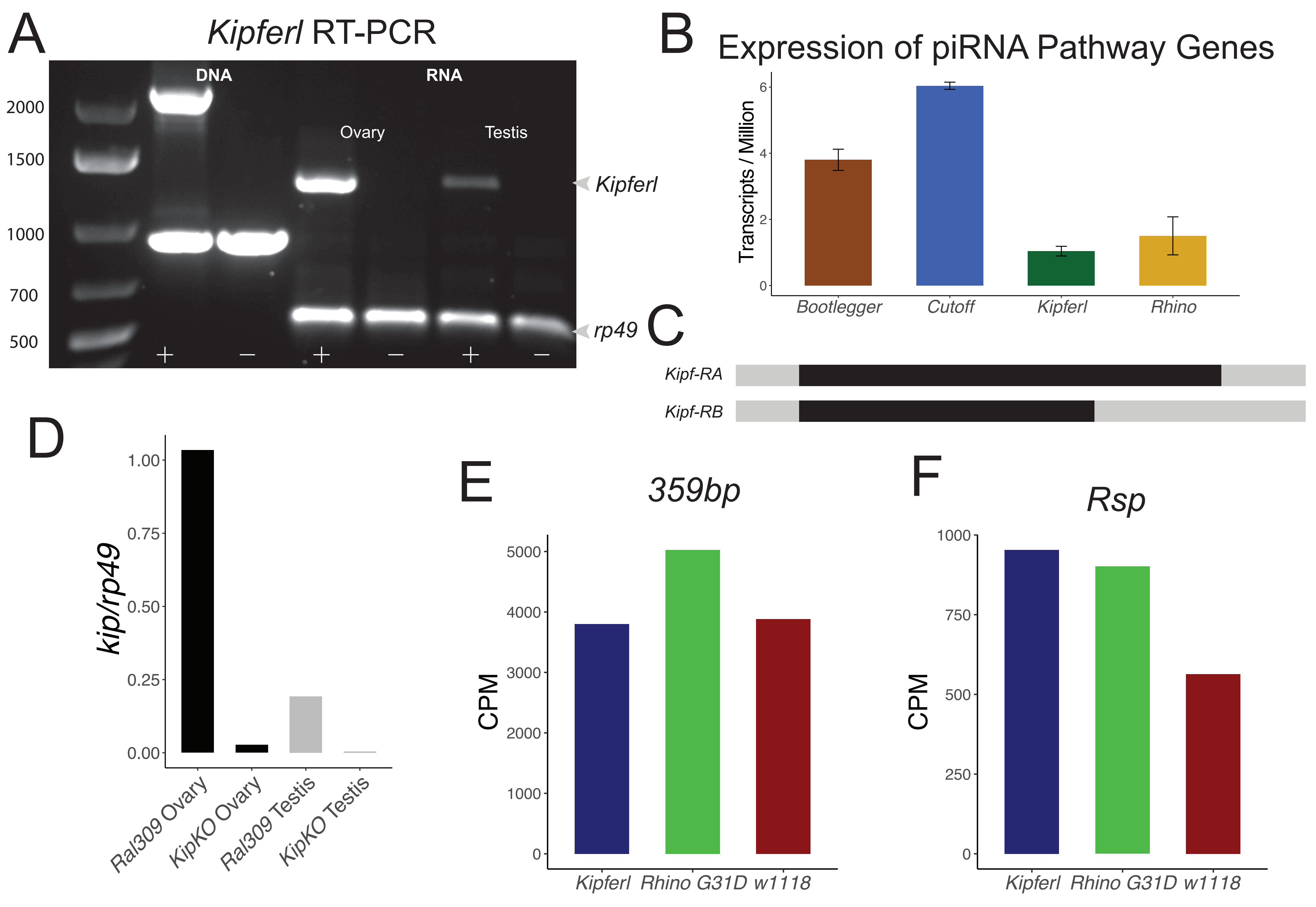
A
Kipferl RT-PCR
DNA
RNA
Ovary
Testis
2000
1500
1000
700
500
Kipferl
rp49
+
−
+
−
+
−
B
Expression of piRNA Pathway Genes
Transcripts / Million
0
2
4
6
Bootlegger
Cutoff
Kipferl
Rhino
C
Kipf-RA
Kipf-RB
D
kip/rp49
0.00
0.25
0.50
0.75
1.00
Ral309 Ovary
KipKO Ovary
Ral309 Testis
KipKO Testis
E
359bp
CPM
0
1000
2000
3000
4000
5000
Kipferl
Rhino G31D
w1118
F
Rsp
CPM
0
250
500
750
1000
Kipferl
Rhino G31D
w1118
