# Supplementary material for: Disruption of small RNAs and mechanistic variation in Segregation Distorter: A sperm-killing drive system in Drosophila melanogaster
Source: PLoS Genet. 2026 Aug 3;22(8):e1012235. doi: 10.1371/journal.pgen.1012235 (PMC13460739; doi:10.1371/journal.pgen.1012235)
Supplement: Supplementary file 12 [file pgen.1012235.s012.pdf]

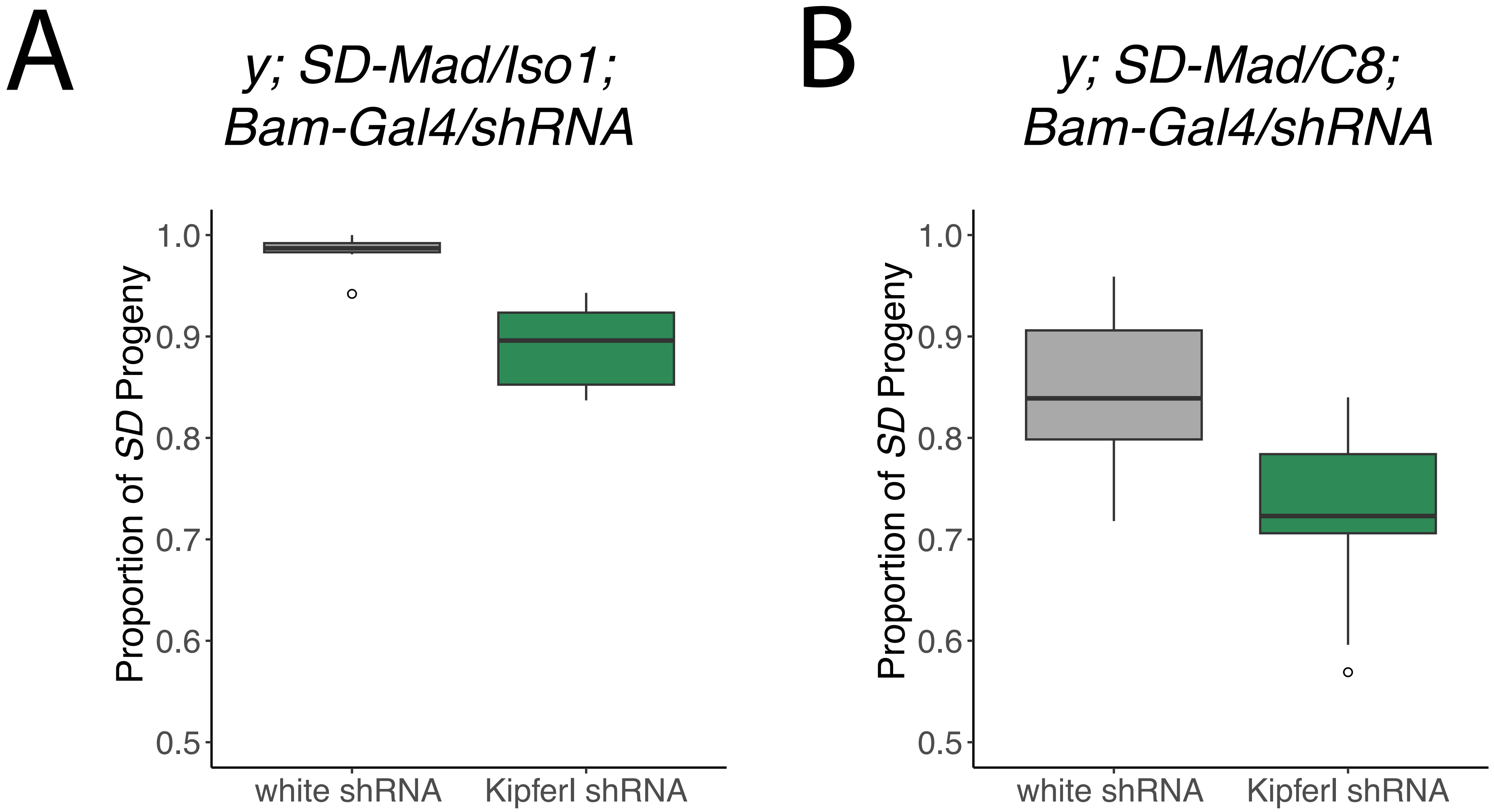
A
y; SD-Mad/Iso1;
Bam-Gal4/shRNA
Proportion of SD Progeny
1.0
0.9
0.8
0.7
0.6
0.5
white shRNA
Kipferl shRNA
B
y; SD-Mad/C8;
Bam-Gal4/shRNA
Proportion of SD Progeny
1.0
0.9
0.8
0.7
0.6
0.5
white shRNA
Kipferl shRNA
