# Supplementary material for: Disruption of small RNAs and mechanistic variation in Segregation Distorter: A sperm-killing drive system in Drosophila melanogaster
Source: PLoS Genet. 2026 Aug 3;22(8):e1012235. doi: 10.1371/journal.pgen.1012235 (PMC13460739; doi:10.1371/journal.pgen.1012235)
Supplement: Supplementary file 13 [file pgen.1012235.s013.pdf]

A

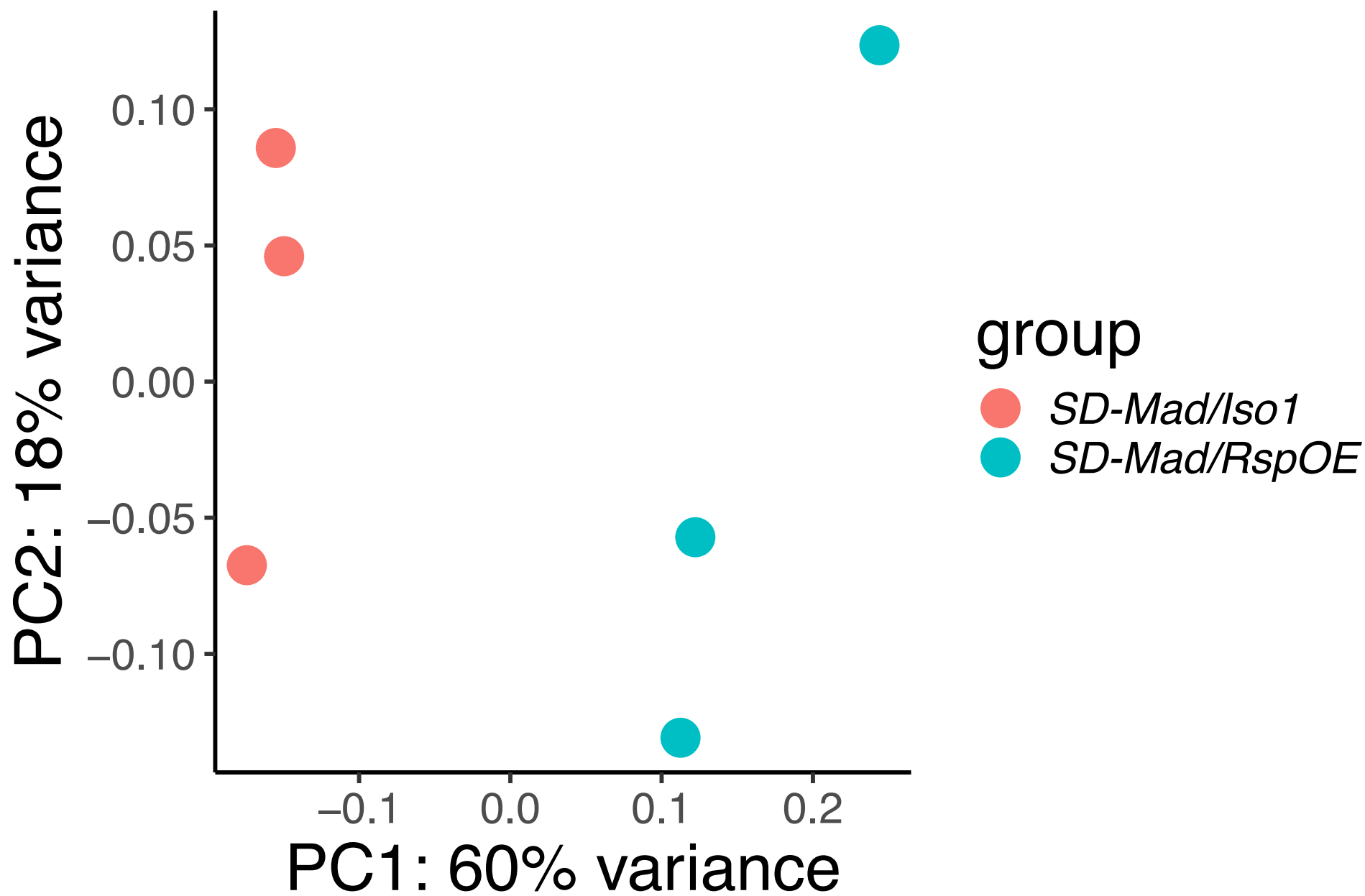

PC2: 18% variance
0.10
0.05
0.00
−0.05
−0.10
−0.1
0.0
0.1
0.2
PC1: 60% variance
group
SD-Mad/Iso1
SD-Mad/RspOE

B

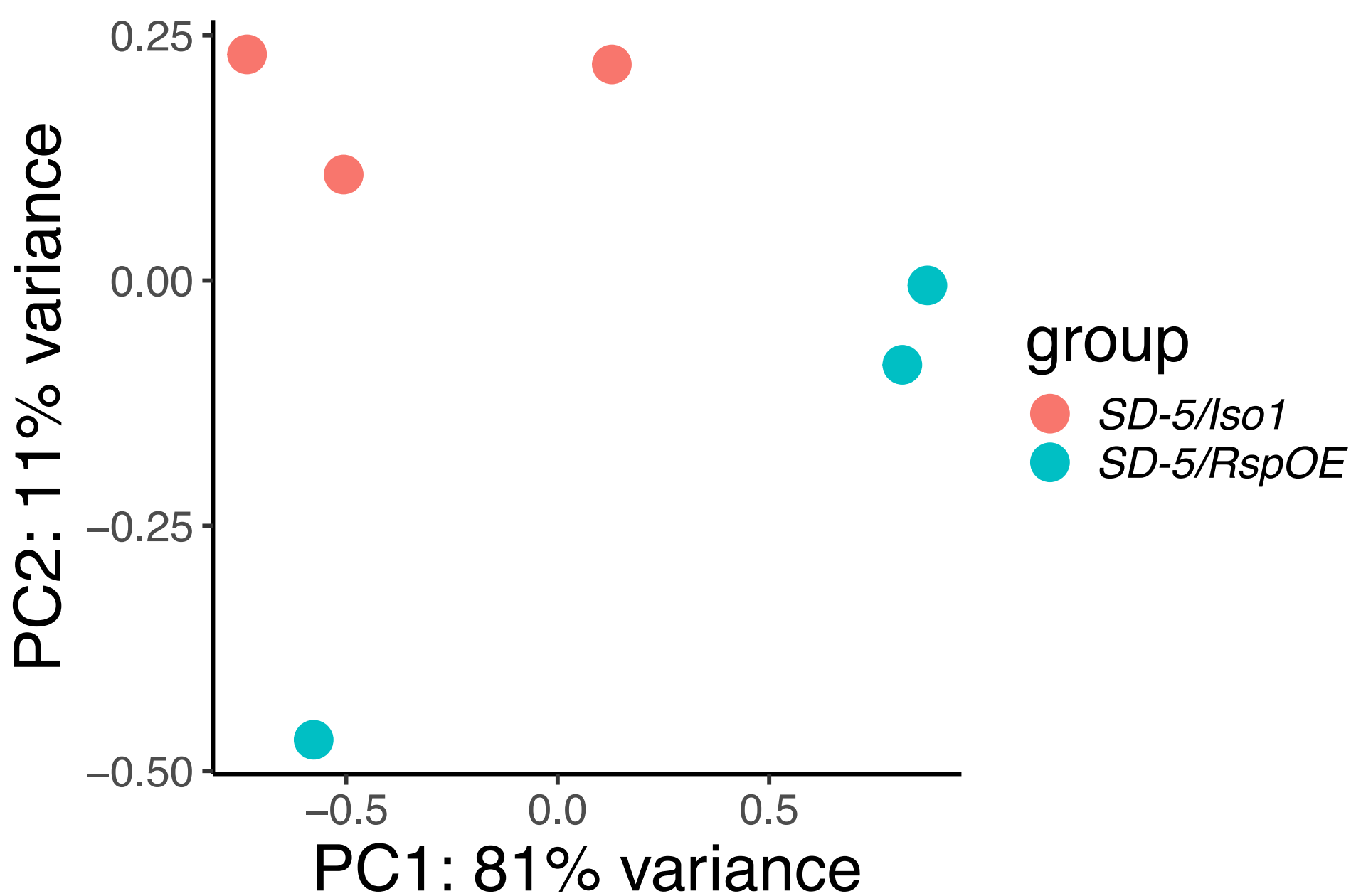

PC2: 11% variance
0.25
0.00
−0.25
−0.50
−0.5
0.0
0.5
PC1: 81% variance
group
SD-5/Iso1
SD-5/RspOE
