# Supplementary material for: Disruption of small RNAs and mechanistic variation in Segregation Distorter: A sperm-killing drive system in Drosophila melanogaster
Source: PLoS Genet. 2026 Aug 3;22(8):e1012235. doi: 10.1371/journal.pgen.1012235 (PMC13460739; doi:10.1371/journal.pgen.1012235)
Supplement: Supplementary file 14 [file pgen.1012235.s014.pdf]

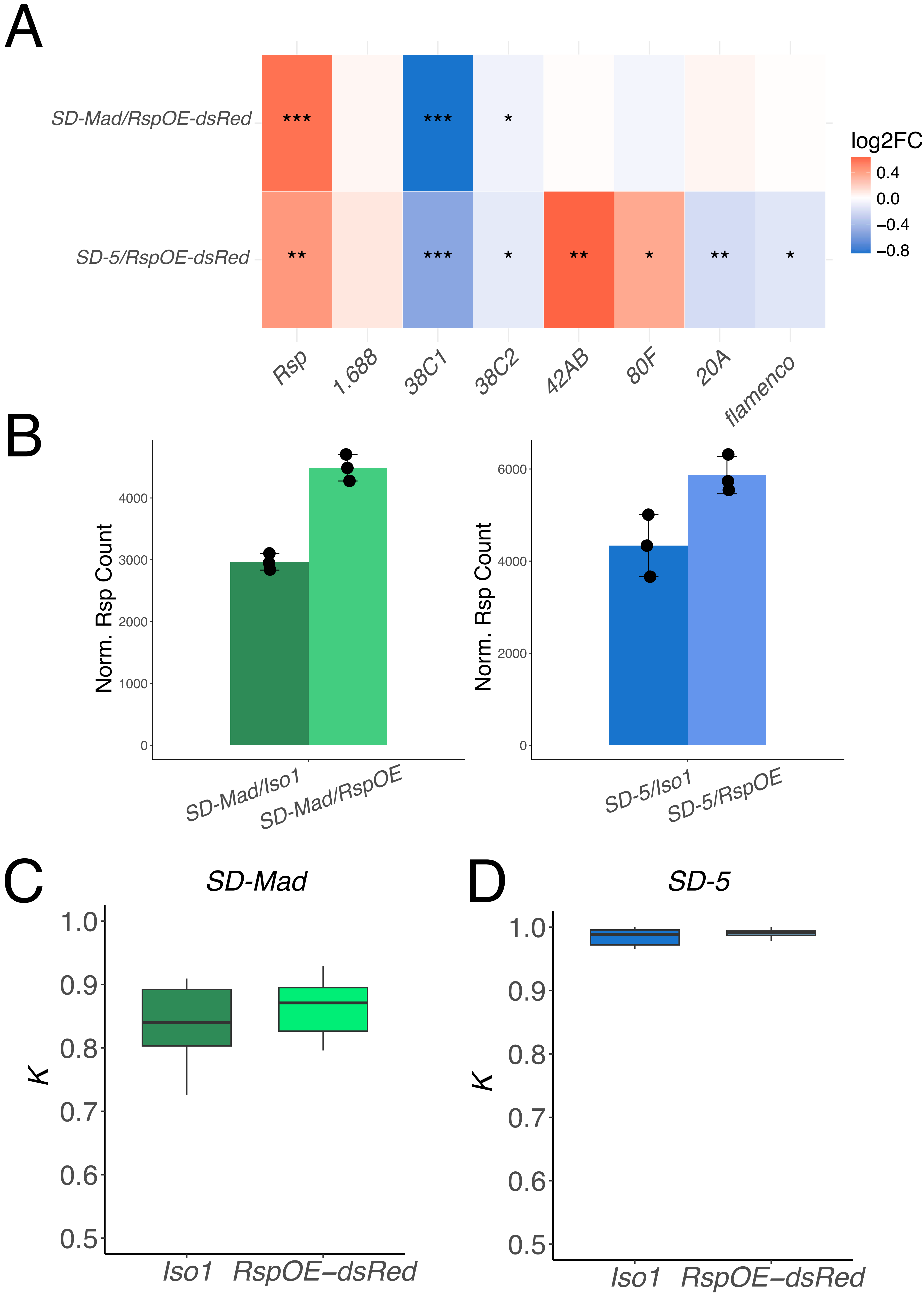
A
SD-Mad/RspOE-dsRed
SD-5/RspOE-dsRed
***
***
*
**
***
*
**
*
**
*
Rsp
1.688
38C1
38C2
42AB
80F
20A
flamenco
log2FC
0.4
0.0
−0.4
−0.8
B
Norm. Rsp Count
0
1000
2000
3000
4000
SD-Mad/Iso1
SD-Mad/RspOE
Norm. Rsp Count
0
2000
4000
6000
SD-5/Iso1
SD-5/RspOE
C
SD-Mad
κ
1.0
0.9
0.8
0.7
0.6
0.5
Iso1
RspOE−dsRed
D
SD-5
κ
1.0
0.9
0.8
0.7
0.6
0.5
Iso1
RspOE−dsRed
