# Supplementary material for: Disruption of small RNAs and mechanistic variation in Segregation Distorter: A sperm-killing drive system in Drosophila melanogaster
Source: PLoS Genet. 2026 Aug 3;22(8):e1012235. doi: 10.1371/journal.pgen.1012235 (PMC13460739; doi:10.1371/journal.pgen.1012235)
Supplement: Supplementary file 15 [file pgen.1012235.s019.pdf]

Full revision template for Review Commons

Manuscript number: RC-2025-03334R

Corresponding author(s): Larracuente, Amanda

1. **General statement:**

We have completed a full revision of our manuscript "Disruption of small RNAs and mechanistic variation in Segregation Distorter—a sperm-killing drive system in Drosophila melanogaster". In this study, we investigate the molecular basis of drive in the *Segregation Distorter* (*SD*) system of *Drosophila melanogaster*, one of the best-characterized examples of male meiotic drive. *SD* biases its transmission by disabling sperm carrying sensitive alleles of its target (+), the *Responder* (*Rsp*) satellite DNA locus. In *SD*/+ males, sperm bearing large numbers of *Rsp* repeats fail to mature properly through a chromatin defect, resulting in transmission of *SD* to more than 90% of offspring. Despite decades of study, the role of the repetitive *Rsp* target in drive has remained unresolved.

Using a combination of transcriptomics, cytology, and molecular genetics, we tested the hypothesis that *Rsp*-derived RNAs contribute to the drive phenotype. Our findings reveal an unexpected role for small RNAs in the regulation of this classic meiotic drive system.

Our work provides new insight into both meiotic drive and satellite DNA regulation during spermatogenesis. First, they establish a direct connection between RNAs complementary to the target satellite and the drive phenotype, extending growing evidence that RNA interference pathways are repeatedly co-opted in diverse drive systems. Second, they uncover mechanistic heterogeneity among *SD* haplotypes, suggesting that interactions between drivers and linked modifiers shape the molecular basis of drive. These results complement population genetic studies that have identified strong epistatic selection acting on *SD* co-adapted gene complexes and provide a mechanistic framework for understanding how such modifiers contribute to the evolution of selfish genetic elements.

This manuscript has been extensively revised following peer review through Review Commons. We received thoughtful and constructive feedback from three expert reviewers and have incorporated their suggestions throughout the revised manuscript. The revision includes new RNA-seq data and analyses, additional clarification of key results, and revisions to the text and figures. Our detailed point-by-point response to all reviewer comments is below.

2. **Point-by-point description of the revisions**

Reviewer #1

Evidence, reproducibility, and clarity

Summary: Edvalson and colleagues use transcriptomics, cell biology and genetics to study variation between segregation distorter (meiotic drive) strains and find several important results. These include apparent suppression of small RNAs mapping to responder (the drive target) in one of the lines, a general pattern of differential expression consistent with the drive mechanism being upstream of sperm individualization (where defects have been seen previously), and genetic confirmation that perturbing Rsp expression can influence the strength of drive.

Major comments: I found the total RNA sequencing experiment a bit oddly presented. This is partly because it was in the middle of the results (might fit better first), partly because few specific genes were discussed (this might be appropriate given then question, but maybe the question should be more clearly stated), and the complexity of the approach (WCGNA + PANGEA) and how it all fits together. I suggest working to clarify the main points of this section (which are a bit different than the main focus of the Rsp work).

We thank the reviewer for these important points. We liked the suggestion to swap the order of our results. We attempted the change, but we found that we weren't able to make the flow of the results much better. Instead, we primed the transition from smRNA to totRNA in the last paragraph of the smRNA results (lines 190-196). This paragraph now reads:

> *The dearth of Rsp smRNAs in SD-Mad heterozygotes could be due to a disruption in transcription of the locus or subsequent processing steps. Many factors can influence piRNA production. For example, the piRNA pathway can amplify piRNAs independently of transcription, such as the ping pong cycle, (Czech and Hannon 2016). Notably, Rsp piRNAs do not have a strong ping pong signature in testes (Wei et al. 2021; Chen et al. 2021a). To distinguish between a disruption in transcription or some downstream process, we examined total RNA.*

The main reason we elected to describe patterns rather than specific genes is that the 2nd chromosomes we tested (*R-16*, *SD-Mad*, *SD-5*) have all diverged from each other and any single differentially expressed gene could be due to differences in genetic background. Therefore, we elected to point out more broad systematic changes in pathways and correlated gene networks rather than specific genes. We have made it more obvious throughout the total RNA section in the text what our question is regarding the transcriptome and the reasoning for using WGCNA and gene set analysis.

We also appreciate the reviewers point that the complex approach we used to extract changes in pathways and networks is difficult to follow. We have modified our wording to better describe the flow of analyses.

We also note that we have extended our analysis for the comparison of *SD-Mad* and *SD-MadRev*, which only differ by the *Sd-RanGAP* locus. Here we do discuss individual genes that are differentially expressed. See below for details about this new analysis.

Minor comments:

Abstract - Probably worth mentioning Sd-RanGAP here, even if you are using it as a straw man. I agree that the specific mechanism is not known, but some of the genetics are established.

This is a good point. While our study doesn't address *RanGAP*, it is important to point out that, although its role in drive is unclear*, Sd-RanGAP* is a necessary component of the system. We added the following language to the abstract:

> *SD is a multigene complex, frequently associated with chromosomal inversions, where the main driver locus, a truncated duplication of the gene RanGAP kills wild-type sperm containing a satellite DNA called Responder (Rsp)*.

Line 80 and elsewhere - it would be helpful to be specific here - you are looking at both small and total RNA

We've modified our wording throughout the manuscript to specify when we are referring to total RNA and small RNA.

Fig 1B - is there a reason not to show the values of the replicates here? It would be more transparent.

We thank the reviewer for this comment. We replaced Fig 1B with a chart that is computed from the DESeq2 normalized counts for each comparison and added replicates to all related graphs.

Add replicate data

Line 139 - does the experimental design control for 1.688 genomic copy number? Where is it located?

We indeed control for the 1.688 copy number here. Most 1.688 repeats are found on the X chromosome and all flies in our experiments have identical X chromosomes. We changed the text to specify that copy number for 1.688 are the same between conditions.

144-146 - this could be written clearer, and I think it should only refer to 1C, not 1B. Part of the issue is that there are several repeats not discussed, and it isn't clear what is happening with them. I suggest expanding this description so it is more clear.

Thank you for this feedback. We have expanded the description to make this section clearer.

Line 161 - what do you mean (specifically) by "repetitive loci"?

Repetitive loci in this case refers to transposons, satellite DNAs (except simple satellites), and piRNA clusters. We have added text explaining what is included the grouping of “repetitive loci”. We have added the following sentence to the text:

> *Our results demonstrate that SD-Mad and SD-5 haplotypes, despite sharing the same main drive locus, have different effects on smRNAs derived from repetitive loci such as complex satellites (including Rsp), transposable elements, and piRNA clusters.*

193-203 - This is an important finding that is somewhat lost in trying to keep track of WCGNA and PANGEA and the different Modules. I suggest clarifying to drive home the point that differential expression appears to start prior to individualization, which suggests and earlier mechanism of drive.

We thank the reviewer for this feedback. We have added wording to out discussion that points out this finding in lines 501-505 which reads:

> *We suspect that the timing of the proximal cause of SD-mediated drive may align with early spermatogenetic processes; perhaps where cell cycle-related genes are active and appear to be broadly differentially expressed (Figure 2B, Module H). This earlier timing is consistent with temperature shift experiments that place the critical period for SD at or before meiosis (Mange 1968)*

Fig 3B & 3C, Fig 4 - same as 1B, is there a reason not to show the actual data points?

A similar issue was brought up earlier, in response we modified all our figures to show replicate points where applicable.

Line ~245 - was the same experiment done with SD-5? (as you do below for Rsp overexpression)

We originally did not include *SD5* in this experiment, but we have since measured drive strength of *SD5* in a kipfKO background. We found a small but statistically significant difference in drive strength. We added the new *SD5* results to the figure and moved the kipfKD data to the supplement along with some added data on a *Rsp* deletion line generated from *Iso1* that bolsters our confidence in the *SDMad* results.

Significance

This is a strong paper that moves the field forward, even if it leaves questions still to be answered (why the difference between drivers? what is the mechanism? how is rsp interacting with drive?

Several findings move the field forward: the Rsp small RNA results, the differential expression hinting at a molecular mechanism that is upstream of sperm individualization.

The audience is moderately broad. Genetic conflict is gaining in general interest, but aspects of this will be mostly interesting to the hardcore drive crowd.

Reviewer #2

Evidence, reproducibility and clarity

I have only one request: I found it unclear whether the authors were referring to small RNAs or their precursor (long RNA). By reading the text carefully, I could deduce that Fig1A/Table S2 represent the small RNA sequencing, while FigS3A represents total RNA seq (detecting precursor). However, the labeling in the Fig1A and Table S2 only says 'piRNA cluster' or 'Rsp' (without clarifying 'piRNA from piRNA cluster' or 'piRNA from Rsp'), and it took quite some time for me to understand which Fig/data is smallRNA vs. longRNA.

This is helpful feedback. We have added more clarity to which type of RNA is being represented in our figures throughout.

Significance

This manuscript by Edvalson et al. describes their study on SD (segregation distorter) meiotic drive system, examining the role of piRNA derived from Rsp satellite. Although the exact mechanism of drive is still unknown, this study represents a significant step forward

in understanding SD-mediated drive.

By using two SD alleles (SD-5 and SD-Mad), they show that Rsp-derived piRNA is depleted in SD-Mad. The authors used total RNA sequencing/small RNA sequencing mutants and carefully designed controls (such as deletion of Sd-RanGAP) to reach the model that Rsp-derived piRNA is involved in SD-Mad-mediated drive. The result that kipferl depletion (that lead to sat DNA expression) rescues SD-Mad's drive phenotype is very interesting. This supports that the decreased Rsp piRNA indeed corresponds to SD-Mad-mediated drive. They further back up this idea by overexpressing Rsp.

Interestingly, SD-5 was not impacted by changes in Rsp expression. Based on this result, the authors state that there are mechanistic variations in the same (SD) drive system. This statement is certainly justified by the data, but I cannot help wondering there might be a unifying mechanism that explains both SD-5 and SD-Mad. I am not suggesting to edit the manuscript or add the discussion: but do they have any speculations on this? For example, SD-5 is simply epistatic to Rsp piRNA production? For example, SD-RanGAP > SD-Mad (some gene on SD-Mad inversion) > Rsp piRNA production > SD-5 > sperm killing?

We thank the reviewer for this insight. We indeed think that the proximal cause of sperm dysfunction could be the same, but there are components of *SD5* that act downstream of *Rsp* piRNAs. The small difference in drive strength in the *SD5* KipfKO experiments might support this hypothesis, although it is also possible instead that drive is influenced by changes in some other piRNAs (from the piRNA clusters or satellites).

We modified our wording in the first paragraph of the discussion to point out this possibility. Lines 367-370 now reads

> *These results suggest that, while SD chromosomes share a target and main drive locus (Sd-RanGAP), the modifiers accumulated on each haplotype may influence the drive mechanisms, either by creating new pathways to drive or acting as tuning knobs on drive strength.*

Reviewer #3 (Evidence, reproducibility and clarity (Required)):

Summary

In the presented manuscript Edvalson and Wei et al use Drosophila genetics and NGS experiments to investigate the mechanism of meiotic drive through the Segregation Distorter (SD) system. They reveal that two driving haplotypes seem to function via different mechanisms, with drive through SD-Mad but not SD-5 involving small RNAs produced from the Responder (Rsp) satellite, the target of SD drive. SD-Mad testes displaying drive are characterized by lower levels of Rsp sRNAs compared to non-drive controls as well as SD-5, and the ectopic overexpression of Rsp sRNAs through two distinct mechanisms decrease drive in SD-Mad genetic background, specifically. With this work, the authors are adding an important piece of information to the highly complex SD system, indicating that sperm killing is likely achieved by different mechanisms in different SD haplotypes, despite sharing a common driver.

Major comments

Fig1C: It might be interesting to show the fold change between SD-Mad and SD-MadRev in addition to what is displayed. Moreover, can the authors comment on what might be causing the increased smRNA counts for 38C2? Is this because R16 has particularly low 38C2 values?

We appreciate the reviewer's comment concerning the fold change between *SD-Mad* and *SD-Mad*^{Rev}. We have made a figure showing the difference between and put it in Figure S1.

We suspect that the expression difference in 38C2 between the *R16* heterozygotes and *SD* heterozygotes may be due to genetic divergence, since these are different 2nd chromosomes. We have added language pointing this out to the manuscript in line 182. The paper now reads:

> *There is no evidence that either 38C2 or Flamenco are involved in SD-mediated drive.*

Fig1/S1: Could the authors also display the Rsp smRNA counts for all Gla crosses similar to panel 1B? What is the interpretation for the increase in Rsp smRNAs in SD-5/Gla relative to R16/Gla but the lack of such an increase in the SD-5/iso1 vs R16/iso1 comparison? Do SD-Mad and SD-5 induce the same strength of drive against each of the two wildtype chromosomes? Experiments: smRNAseq for SD-MadRev/Gla.

We have added a plot to Fig S1 to show the abundance of *Rsp* small RNAs in the *Gla* background, similar to Figure 1.

It is difficult to interpret the apparent overabundance of *Rsp* small RNAs in the *SD-5/Gla* background. Because differences in *Rsp* smRNA abundance for *SD-5* are inconsistent between the *Iso1* and *Gla* background, our interpretation is that *SD-5* is not manipulating *Rsp* levels. The apparent overabundance of *Rsp* in the *Gla* background could be due to an epistatic interaction between *Rsp* and other components of that particular background. Consistent with this interpretation, the *SD-Mad* induced reduction of *Rsp* smRNAs in the *Gla* background is less dramatic than in the *Iso1* suggesting that something about that background is increasing *Rsp* expression slightly when paired with an *SD* chromosome.

Fig1: The authors note changes in smRNA levels for other satellites as well as piRNA clusters but do not give any interpretation to this observation. Are they meaningful? Should they be attributed to genetic background?

Our interpretation of the observation that some satellites or piRNA clusters are differentially expressed is that these differences are likely due to epistatic effects from the different 2nd chromosomes used in the study or are incidental to mechanism of *SD*.

FigS2: Same question also for the deregulated TEs: do they share sequence features with Rsp or are they overrepresented in the clusters that change? Are these explained by differences in insertions between genotypes? Do their total RNAseq values change in any way? What do the percentages in line 162 correspond to? Number of TEs that are deregulated? At which cutoff? It might be informative to compare the data to a cross between driver and R16, or even better the SD-MadRev control. Experiments: totRNAseq for SD-MadRev crosses and optionally crosses to R16.

The *Rsp* repeat unit does not share significant homology to portions of the genome outside of the pericentromere of 2R with the exception of ~6-12 copies in the intron of *Ago3*.

As far as TEs are concerned, we surprisingly don't see a strong correlation between piRNA cluster content, dysregulation, and TE transcript abundance. For example, in the *SD/Gla* backgrounds the total RNA for *R1*, *R2*, *IGS*, and *Tc1-Mariner* family TEs is down regulated. However, the only major piRNA cluster that is upregulated in both *SD/Gla* backgrounds (*80F*) is not enriched for TE fragments matching any of those 4 families. One thing we can note is that the definition of the major piRNA clusters are given in relation to the *Iso1* genome which may differ from that of our experimental backgrounds. Without long read resolved genomes for our specific experimental lines generated at the same time as the RNA samples it is difficult to determine how expression at the major piRNA clusters and the corresponding TEs are related. We have described this lack of a correlation in lines 210-217 in the text along with our interpretation for why this could be. The paper now reads:

On the other hand, we did find some differences in repetitive elements related to rDNA (R1, R2, and IGS) and Tc1-Mariner family TEs (all backgrounds; Figure S6). Interestingly, there was no correlation between the expression of TEs and the expression of piRNA clusters that contain fragments of these TEs in the total RNA, nor was there any correlation between the small RNAs from piRNA clusters and the total RNAs for those TEs. PiRNA clusters are usually defined in one isolate of Iso1: rapid turnover of TEs and piRNA sources could explain why we do not see a correlation between piRNA cluster expression and TE expression in our backgrounds.

We investigated differences in TE and piRNA cluster expression in our *SD-Mad/Iso1* vs *SD-MadRev/Iso1* comparison, but a lack of power due to inter-sample variation prevents us from confidently making any assessments on any TEs or piRNA clusters in that comparison. We did however generate additional gene level transcriptomic data using 3' Digital Gene Expression to bolster our confidence in the totRNA data and found some interesting genes that were in the top most differentially expressed. We have noted those genes in lines 276-287 which read:

To identify genes that might interact to cause drive, we compared the gene expression of SD-Mad/Iso1 to SD-MadRev/Iso1. These genotypes only differ by the presence of the main drive locus, Sd-RanGAP. We performed both totRNA and 3' Digital Gene Expression (DGE) RNA sequencing and examined the overlap in differential expression between the totRNA and DGE sequencing. There are 69 differentially expressed genes where the DGE comparison is significant (PDGE ≤ 0.01), and the sign of the Log2FC of the totRNA matches that of the DGE. Among this set of differentially expressed genes, 57 show at least a 50% difference in gene expression (absolute Log2FC value of at least 0.58 in DGE). These genes are not enriched in any Reactome gene sets. The top 20 most differentially expressed genes consists of 9 lncRNAs (3 anti- sense RNAs) and 11 protein coding genes: 8 of which are uncharacterized. The 3 characterized genes are Artemis (Arts), Gr61a, and Tono (Figure S98, Supplemental File 1).

We discuss two of these genes in further detail in the discussion in lines 476-486 which read:

First, Tono, a BTB zinc finger-containing transcription factor is upregulated (Log2FCDGE = 1.7) in all SD-Mad comparisons. Tono plays a role in regulating transcription in muscle cells in response to mechanical pressure (Zhang et al. 2024) but also shows enrichment in male germ cells (Li et al. 2022). The putative DNA-

binding capacity and ability to form nuclear condensates (Zhang et al. 2024) makes this an interesting candidate gene for interacting with the Rsp satellite. Second, the importin-4 ortholog, Artemis (Arts), which facilitates Ran-mediated import of H3 and H4 is overexpressed in SD-Mad (Log2FCDGE = 2.5). Interestingly, Arts expression is antagonistic to male fertility (VanKuren and Long 2018). Also of note, Apollo, a duplicate of Arts which supports male fertility (VanKuren and Long 2018) is downregulated (Log2FCDGE = -0.6) though it is not in the top-most differentially expressed genes.

Figure S3: Am I reading the PCA plots right in that there are very few gene expression changes when the drivers are in iso1 background but much more in the Gla background? Comment on possible explanations for that. Please indicate the number of significantly changed genes in each comparison. Again, are these changes correlated between the two drivers or can they be attributed to genetic background of Gla vs R16? Would it be interesting to see how SD-Mad/Gla and SD-5/Gla gene expression profiles compare? Experiment: totRNAseq for SD-MadRev crosses.

There did tend to be more differences in the *Gla* background compared to *Iso1*. This difference can best be explained by inter-sample variation in the *SD-Mad/Iso1* background which we see in the PCA plot in Fig S4A. Another reason for the difference could be that the *Gla* and *Iso1* chromosomes are very different from each other which prevents us from making any 1-to-1 comparisons between the *SD/Iso1* and *SD/Gla* backgrounds. We generally avoid comparing between genetic backgrounds for this reason unless they share differences as these are more likely related to drive.

In Figure S5A it seems that totalRNA levels of Rsp are strongly increased in SD-Mad/Gla but not in SD-Mad/iso1. The iso comparison (less piRNAs but same transcript) could indicate that it is actually transcription of the Rsp that is affected here. This is even pointed out in line 205 without discussion of the fact that the Gla comparison (less piRNAs but more transcript) would rather indicate that transcription is intact, but processing into piRNAs is defective. Could this be clarified using FISH as in Figure S8? If true, SD-Mad/Gla should have much more FISH signal than SD-Mad/iso1. Either way, this discrepancy should be further discussed. Experiments: comprehensive smFISH panel for all crosses (including SD-MadRev).

The reviewer makes an excellent point. Why would *Rsp* long RNAs be overexpressed in the *SD-Mad/Gla* background? Earlier we noted that in the *Gla* background specifically the genotypes that contain an *SD* chromosome seem to have a higher level of *Rsp* small RNAs than we might expect given our *Iso1* results. We conclude that this is likely due to an

epistatic interaction between the 2nd chromosomes used in the study and the rest of the chromosomes. This interpretation could extend to the long noncoding precursors as well.

Further, although the difference between *SD-Mad/Gla* is significant and *SD-5/Gla* is not, they do move in the same direction. This is also true in the *Iso1* backgrounds but in the opposite direction. Given an interpretation that *Rsp* expression is higher than expected in the *SD/Gla* background due to epistatic effects, it becomes clearer that changes in long RNA abundance are related to changes in small RNA abundance though not perfectly indicative. However, due to lower count levels for *Rsp* in the totRNA, we do not have the power to confidently draw that conclusion.

In general, the totRNA profiles of repeats don't seem to correlate well between the genotypes (iso vs Gla crosses, neither for SD-5 nor for SD-Mad). Is this because values are in general small and/or replicates don't correlate? Should these data even be considered? Also panels 2A and S5C are very different from each other. The additional comparison with the SD-MadRev allele crossed into both Iso1 and Gla should give additional insight. Experiment: totRNAseq for SD-MadRev crosses.

The reviewer brings up a good point. While some repetitive elements had relatively small counts in the totRNA (like *Rsp*) most had adequately high counts. But these differences are to some degree expected. Although the other chromosomes are controlled for, the second chromosomes are different by design including the two *SD* haplotypes. In this context, similarities between the two haplotypes may be helpful in determining some unifying aspects of the *SD* mechanism but differences could be incidental to the genotype and not necessarily related to *SD*.

It may be generally informative to set the sRNA and RNA comparisons into perspective, for example by including the comparison of SD-Mad crosses versus SD-MadRev crosses to exclude unrelated genetic background components as much as possible.

The reviewer is correct here. Differences in the transcriptomes of *SD-Mad* and our revertant are much more likely due to the drive phenotype. Due to variation between *SD-Mad* total RNAseq replicates, we have substantially less power when comparing *SD-Mad/Iso1* to *SD-Mad^{Rev}/Iso1*. We therefore generated new data to address this point: we did digital gene expression for three biological replicates of *SD-Mad/Iso-1* and *SD-Mad^{Rev}/Iso1*. We described the results of this new analysis above.

FigS6: I assume this is given, but as it is not specified: is the directionality of differential expression taken into account here? Or could it be significantly up in one and down in the other? Please specify / adjust color scale to allow this distinction.

This is a good point. We have modified the figure to not only indicate significance but also direction and magnitude.

FigS8: Please add a scale bar for all images. 1.688 is labeled as 359 in the legend, please unify or/and explain nomenclature. Consider adding a nuclear outline based on DAPI. It looks like 1.688 is actually more different between control and SD-Mad/Iso than Rsp. Could the authors comment on this? In the text the authors mention that these experiments were done for both SD-Mad and SD-5 heterozygotes, but only the SD-Mad data are shown.

The most abundant component of *1.688* repeats is the *359bp* repeat which is used as a proxy for *1.688* and our 359-bp probe cross hybridizes with other abundant variants of 1.688 on chromosome 3. We agree, there does seem to be some differences in the *1.688* RNA FISH, however we do not yet have evidence that *1.688* is related to the drive phenotype. We have expanded that figure (now supplemental figure 7) with multiple images for each genotype to demonstrate the lack of change in *Rsp* and 1.688 localization. We have added an explanation of the nomenclature.

The reference to *SD-5* in the text was made in error. We do not have RNA FISH images of *SD-5/Iso1* heterozygotes. We've modified the text to reflect this.

FigS9B: What does the y-axis label mean? Fold change relative to what? Is this not displaying counts?

This is a good catch by the reviewer. The y-axis is mislabeled and should read "TPM". We have made this change.

To set the KipfKD/KO data in context, please give also the k value for SD-MadRev and compare the smRNA values in this context to the data displayed in F1B. Experiment: drive analysis for SD-MadRev.

Our basis for concluding that *Rsp* smRNA overexpression may reduce drive strength is in demonstrating that kipfKO is sufficient to rescue wild type sperm in driving backgrounds. We did not introduce KipKD (or KO) to the *SD-Mad*^{Rev} background because this chromosome does not drive.

The note that the 3XP3-dsRed cassette needs to be flipped out for Rsp overexpression to influence drive is interesting. It would be great if the authors could show a more detailed scheme of the structure of this insertion including the directionality of the promoter relative to the Rsp fragment and the rest of cluster 38C (including dm6 coordinates perhaps). Small RNA sequencing compared to totRNA sequencing should reveal if the transcription or the processing into piRNAs of the inserted piece is affected, and if more of the 38C piRNAs are affected. Genic transcription has been previously observed to limit Rhino-dependent

piRNA production from piRNA clusters (Andersen et al 2017). It might be of interest to the general piRNA community to see how cluster output is influenced through the integration of an internal genic promoter.

We agree that this is an interesting result. We have added more detail to Fig 4A to indicate directionality and genomic location of the insert in terms of dm6.

Figure panel 4A should be adjusted to include annotations of the black boxes and to give genomic locations. It is unclear what the blue brackets mean, and where exactly the insertion took place. Are the attP sites relevant for the experiments? It might be nice to see a piRNA profile over the locus, to put the levels of additional Rsp piRNAs into perspective.

We have removed the black boxes from the schematic as they were only there as an aesthetic choice. We have indicated where exactly the insertion was made. The attP sites are there for future experimental flexibility.

Minor comments

Figure 3B: fold change of satellite RNA is shown. It might be obvious that the fold change relates to KipfKO / WT but this should be stated explicitly. What is the genetic background here?

Thank you for the comment. We added information on the genetic background in the figure.

Figure legends should be extended for clarity throughout the manuscript in main and supplementary figures. All color codes and abbreviations as well as samples / genotypes and assay used should be clearly explained. Few examples include: F1B: smRNA or totalRNA? F3B: fold change relative to what? F4B: what are these data relative to? F4C: smRNA or totalRNA? S2: Is this smRNAseq? Further description of the color code in the volcano panels would be desirable. FS3: typo in A-B should be A-D. Fold changes relative to what. Etc.

Thank you for these helpful suggestions. We have edited the figure legends as suggested to improve the clarity. We appreciate the feedback.

The abbreviation for Kipferl is kipf, not kip.

Thank you for pointing this out, we have made the corrections.

I don't understand the sentence on lines 310-312.

We agree that sentence was confusing. We replaced it with:

"Identifying potential proteins that interact with Rsp may therefore provide important clues about why satellites like Rsp are targets of drive."

Referee cross-commenting

I agree with the other reviewer's assessments

Reviewer #3 (Significance (Required)):

General assessment

This study of a highly complex and poorly understood drive system adds a very interesting piece to the puzzle of understanding the interplay between a RanGAP duplication and a large satellite array. It's strengths lay in the use of genetics tricks to modify drive (SD-MadRev allele, KipfKO, Rsp cluster insertion). The main weakness of the study is the relatively low correlation of several observations between drive crosses to the Iso1 and Gla lines and lack of explanations thereof. Neither gene nor repeat expression seem to give a convincing overlap in any direction.

Furthermore, it is interesting that SD-Mad and SD-5 have such different dependencies on Rsp sRNA. While outside the scope of this work, it would be very interesting to see how other drive haplotypes behave: is SD-5 the exception or is it SD-Mad (as the authors have also wondered in the discussion). Such additional comparisons may clarify also the discrepancies in RNAseq.

Advance

While it has been previously shown by the same group that Rsp satellites give rise to smRNAs through the piRNA pathway, it is to my knowledge unclear how and if these smRNAs influence drive. This study thus presents a conceptual advance in that it demonstrates that the role of Rsp smRNAs is not shared among driving haplotypes.

Audience

This study is relevant for a highly specialized audience interested in meiotic drive. It contributes to the understanding of the SD system and may serve as a basis for future research in this area. In addition, results reported in Figure 4 may be of peripheral interest for the Drosophila piRNA community for technical interests.

This reviewers expertise: Drosophila, piRNA pathway, heterochromatin, sRNA

This reviewers limitations: nuclear-cytoplasmic trafficking, cytoskeleton
